# Improved prognosis for elderly patients with metastatic renal cell carcinoma in the era of targeted therapy

**DOI:** 10.3892/mco.2021.2317

**Published:** 2021-06-10

**Authors:** Jun Teishima, Daiki Murata, Shogo Inoue, Tetsutaro Hayashi, Koji Mita, Yasuhisa Hasegawa, Masao Kato, Mitsuru Kajiwara, Masanobu Shigeta, Satoshi Maruyama, Hiroyuki Moriyama, Seiji Fujiwara

Mol Clin Oncol 12:557-564, 2020; DOI: 10.3892/mco.2020.2020

Following the publication of the above article, the authors have requested a change in the authorship on the paper. The final author in the author list, Professor Akio Matsubara (of the Department of Urology, Graduate School of Biomedical and Health Sciences, Hiroshima University), contacted the Editorial Office to say that he should not have been listed as an author in this published article. His inclusion as an author on the paper arose as a misunderstanding on the part of some of the other authors.

The revised authorship on the paper is shown above, now excluding Akio Matsubara. All the remaining authors agree with the revision made to the authorship on this paper, and the authors remaining on the paper apologize for any inconvenience caused. Therefore, the revised authors’ names and affiliations in this paper are as follows:

Jun Teishima^1^, Daiki Murata^2^, Shogo Inoue^1^, Tetsutaro Hayashi^1^, Koji Mita^2^, Yasuhisa Hasegawa^3^, Masao Kato^4^, Mitsuru Kajiwara^5^, Masanobu Shigeta^6^, Satoshi Maruyama^7^, Hiroyuki Moriyama^8^ and Seiji Fujiwara^9^

^1^Department of Urology, Graduate School of Biomedical and Health Sciences, Hiroshima University, Hiroshima 734-8551; ^2^Department of Urology, Hiroshima-City Asa Citizens Hospital, Hiroshima 731-0293; ^3^Department of Urology, Fukuyama Medical Center, Fukuyama, Hiroshima 720-8520; ^4^Department of Urology, Hiroshima General Hospital, Hatsukaichi, Hiroshima 738-8503; ^5^Department of Urology, Hiroshima Prefectural Hospital, Hiroshima 734-8530; ^6^Department of Urology, Kure Medical Center and Chugoku Cancer Center, Kure, Hiroshima 737-0023; ^7^Department of Urology, Miyoshi Central Hospital, Miyoshi, Tokushima 728-8502; ^8^Department of Urology, Onomichi General Hospital, Onomichi, Hiroshima 722-8508; ^9^Department of Urology, Higashi-Hiroshima Medical Center, Higashi-Hiroshima, Hiroshima 739-0041, Japan

